# Flexible Amperometric Immunosensor Based on Colloidal Quantum Dots for Detecting the Myeloperoxidase (MPO) Systemic Inflammation Biomarker

**DOI:** 10.3390/bios13020255

**Published:** 2023-02-10

**Authors:** Yanbing Tao, Yunong Zhao, Le Wang, Jing Huang, Yan Chen, Qing Huang, Boxiang Song, Hua-Yao Li, Jianjun Chen, Huan Liu

**Affiliations:** 1 School of Integrated Circuits, Wuhan National Laboratory for Optoelectronics, Optics Valley Laboratory, Huazhong University of Science and Technology, Wuhan 430074, China; 2 CHINALLERGY Biotech Co., Ltd., Wuhan Institute of Biotechnology, 666 Gaoxin Road, Wuhan 430079, China; 3 Wenzhou Institute of Advanced Manufacturing Technology, Huazhong University of Science and Technology, Wenzhou 325000, China; 4 Department of Otorhinolaryngology, Union Hospital, Tongji Medical College, Huazhong University of Science and Technology, Wuhan 430022, China

**Keywords:** myeloperoxidase, colloidal quantum dot, flexible immunosensor, inflammatory disease, amperometric sensor

## Abstract

Myeloperoxidase (MPO) has been demonstrated to be a biomarker of neutrophilic inflammation in various diseases. Rapid detection and quantitative analysis of MPO are of great significance for human health. Herein, an MPO protein flexible amperometric immunosensor based on a colloidal quantum dot (CQD)-modified electrode was demonstrated. The remarkable surface activity of CQDs allows them to bind directly and stably to the surface of proteins and to convert antigen–antibody specific binding reactions into significant currents. The flexible amperometric immunosensor provides quantitative analysis of MPO protein with an ultra-low limit of detection (LOD) (31.6 fg mL^−1^), as well as good reproducibility and stability. The detection method is expected to be applied in clinical examination, POCT (bedside test), community physical examination, home self-examination and other practical scenarios.

## 1. Introduction

Myeloperoxidase (MPO) has been demonstrated to be a biomarker of neutrophilic inflammation in various diseases [1,2,3]. Relevant studies have shown that the concentration level of MPO can reflect the degree of cardiovascular health [4,5] and inflammation in the body. For instance, patients with MPO concentrations greater than 350 ng mL^−1^ in their serum are at increased risk for cardiovascular disease. Moreover, MPO extracted from plasma of rheumatoid arthritis patients also showed significantly higher concentration levels (ng mL^−1^) than those of healthy controls. Despite the different requirements of different diseases, concentration range measurements are pmol L^−1^ (pg L^−1^)–nmol L^−1^ (ng L^−1^) [6,7]. Therefore, low concentration and accurate MPO detection is of great significance. Generally, Enzyme-linked immunosorbent assay (ELISA) and Western blotting are utilized to achieve antibody-based MPO immunodetection. Both assays above usually take several hours or more because of multiple sample processing procedures conducted in the laboratory [8,9,10,11]. Moreover, difficulties in quantitative analysis arise in Western blotting [12]. Therefore, there is an urgent need to develop a more accurate and efficient method for quantitative analysis of MPO.

The principle of electrochemical amperometric immunosensors is based on the electrical properties of the device to track the charge transfer caused by the antigen–antibody specific binding reaction of the biomarker. It can directly convert biomolecular recognition information into electrical signals of sensors which have been used in MPO detection in recent years. Amperometric immunosensors can detect MPO in plasma, and the electrodes need to be cleaned for reuse during the detection process [13]. In addition, in order to improve the detection sensitivity, carbon nanotube wiring was used to amplify the signal of the electrochemomagnetic immunosensor detected by MPO, and the detection time was 30 min [14]. In order to balance the cost and detection speed, it is necessary to explore the mechanism of interfacial charge transport between biomolecules and electrodes in biosensitive layers.

Carbon quantum dots are widely used due to their cleanliness and low potential toxicity. However, such use is also limited to the practical application in the field of electrochemical biosensors due to the low quantum yield, small specific surface area and poor crystallinity [15,16]. In contrast, colloidal quantum dots (CQDs), as a kind of zero-dimensional semiconductor nanocrystal, exhibit typical diameters ranging from 2 to 20 nm [17]. Use of the CQDs has gradually become a novel research strategy in the field of biosensing in recent years [18]. CQDs have a very large surface area, many dangling bonds, and therefore abundant active sites. Protein molecules can be stably bonded to CQDs by ligand exchange [19,20]. In addition, the unique quantum confinement effect and quantum tunneling effect endow CQDs with outstanding functions in biomolecule recognition and electrical signal transduction [21]. CQDs also have great solution processability and compatibility with various rigid and flexible substrates, which ensures strong manufacturability for the sensors. More surprising is that recent research shows that the newly synthesized quantum dot material has the characteristics of low toxicity, which has the potential to be applied in clinical use [22,23].

Based on the functions of CQDs mentioned above, our previous work proposed a method to label protein molecules using CQDs and realized the specific detection of related disease biomarkers at low concentrations. In the previous study, an all-solid CQD-modified sensor was designed, which realized the detection of SARS-CoV-2 IgG antibody and antigen at an ultra-low concentration (ng mL^−1^) [24]. The relevant theoretical results were also verified by detecting cytokeratin 18 (CK18, bladder cancer biomarker) [25]. On the other hand, relevant studies have confirmed that eosinophil cationic protein (ECP) and MPO can be used as local markers of nasal inflammation. Quantitative detection of ECP in actual nasal secretion was achieved in the previous study [26]. The combined detection of ECP and MPO will provide a key basis for the classification of different nasal inflammations.

In this paper, we propose a flexible CQD-modified MPO protein immunosensor which can convert the specific binding reaction between antigen and antibody into a significant electrical current signal. The flexible immunosensor provides quantitative analysis of MPO protein with an ultra-low limit of detection (LOD) (31.6 fg mL^−1^), as well as good reproducibility, repeatability, and stability. It is promising for future applications in portable inflammatory biomarkers detection and wearable health monitoring.

## 2. Materials and Methods

### 2.1. Reagents

Materials for lead sulfide CQD synthesis, including lead oxide, oleic acid, octadecene, hexamethyldisilane, toluene, acetone, and n-octane, were obtained from Aladdin Reagent Co., Ltd. (Shanghai, China). Bovine serum albumin (BSA) with 99.5% purity was provided by Shanghai Aladdin Biochemical Technology Co., Ltd. (Shanghai, China). MPO antigen and MPO antibody were provided by AtaGenix Technology Co., Ltd. (Wuhan, China) and CHINALLERGY Biotechnology Co., Ltd. (Wuhan, China), respectively. Phosphate-buffered saline (1 × PBS, PH = 7.4) used in the experiments was purchased from GE Healthcare Life Sciences Hyclone Laboratories (Logan, UT, USA).

### 2.2. Instruments

The surface characteristics of the device samples were measured by scanning electron microscope (SEM, GeminiSEM300, Carl Zeiss, Jena, Germany). Fourier transform infrared spectroscopy (FTIR) was used to analyze the changes of functional groups at the micro level (VERTEX 70, Bruker, Billerica, MA, USA). All electrochemical experiments were performed in an electrochemical workstation (CHI760E, Shanghai Chenhua, Shanghai, China).

### 2.3. Fabrication of PbS CQDs-Modified Electrode

**Figure 1:**
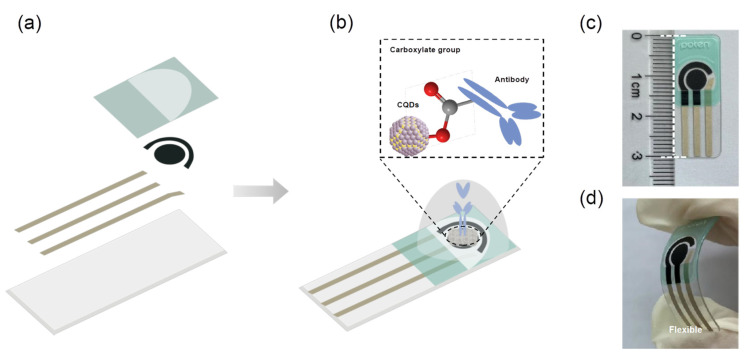
Fabrication of the CQD-modified electrode as the MPO protein immunosensor. (**a**,**b**) Schematic diagram of the preparation process for immunosensor; (**c**,**d**) Optical images of the flexible CQD-modified electrode.

A schematic diagram of the immunosensor fabrication process in this work is shown in Figure 1a,b. Qingdao Porteng Technology Co., Ltd. provided the planar electrochemical electrodes required in this experiment. The electrode size is 30 mm in length, 12 mm in width, and 0.5 mm in height (Figure 1c). The working electrode (WE) and counter electrode (CE) were made from carbon, while the reference electrode (RE) was made from silver/silver chloride. Specifically, the electrode base was made of polyethylene terephthalate (PET) for good device flexibility (Figure 1d).

The specific preparation process of the electrochemical immunosensor for MPO detection was illustrated as the following: PbS CQDs were synthesized according to the literature [19]. Then, 1 μL of lead sulfide solution (10 mg mL^−1^) was dripped onto the WE surface, which can be dried naturally at room temperature to form a modified layer of CQDs. Then, 2.5 μL of MPO antibody protein at 3.96 mg mL^−1^ concentration was carefully added to the solid membrane of CQDs, using a pipette. This process required one hour incubation at 37 °C for complete MPO antibody protein adsorbance to the surface of CQDs. The coated electrode was then blocked with 200 μL BSA solution at 10 mg mL^−1^ concentration in order to passivate the non-specific binding sites on the electrode surface. Under ambient conditions, the electrode surface was rinsed with PBS buffer after one hour blocking. After drying at room temperature, a lead sulfide quantum dot-modified MPO antibody protein electrode was obtained.

### 2.4. Electrochemical Measurements

Cyclic voltammetry (CV) and differential pulse voltammetry (DPV) measurements were utilized to explore the basic electrochemical properties of the modified electrode samples. CV and DPV were performed on bare carbon electrode (CE), CE/CQDs CE/CQDs Antibody and CE/CQDs/Antibody/BSA by dropping a certain amount of PBS as a REDOX medium solution onto the electrode surface. Furthermore, the variations in the electrochemical properties of the electrode during the lamellar modification were investigated. The differential pulse voltammetry characteristics of MPO antigen solution with different concentrations on the WE surface were tested. Then, variation law guiding the device output signal versus the MPO concentration was analyzed. The reproducibility of the MPO measurements was examined by using five parallel electrodes at 10 ng mL^−1^ concentration. The repeatability of the experiment was performed by five measurements of one electrode at a concentration of 10 ng mL^−1^ MPO. For the stability experiment, the electrode was stored in a refrigerator at 4 °C and the performance of the electrode was retested after 9 days. The specificity of the sensor was verified by measuring its response to the possible interfering substances such as non-fat dry milk (NFM), ovalbumin (OVA) and human normal IgG. The relevant detection experiments were carried out at room temperature of 26 °C, and the test condition was PH neutral (7.4).

## 3. Results

### 3.1. The Characterization of PbS CQDs-Modified Electrode

**Figure 2:**
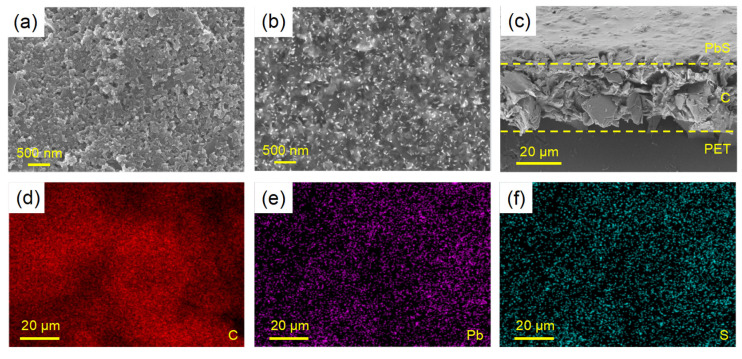
(**a**) SEM image of PbS CQDs film on the work electrode (WE), revealing uniform morphology; (**b**) SEM image of BSA-blocking PbS CQDs film on the work electrode; (**c**) Cross-sectional SEM image of PbS CQDs film on the WE; (**d**–**f**) EDS spectra of PbS CQDs film on the WE.

More details in the morphology of CQD solids film were characterized via scanning electron microscopy (SEM). Figure 2a,b show the surface morphologies of the lead sulfide-modified working electrode sample and the protein-coated working electrode sample, respectively. Figure 2c is the sectional view of the working electrode SEM image. It can be seen that the surface of the CQD-modified layer is flat. The gaps and holes on the CQD film show a regular arrangement. The number of gaps and voids on the membrane surface decreased after protein coating. The cross-section image shows that the thickness of the CQD-modified layer is uniform. Energy dispersive spectroscopy (EDS) showed that lead and sulfur elements on the surface of the modified electrode were evenly distributed (Figure 2d–f).

**Figure 3:**
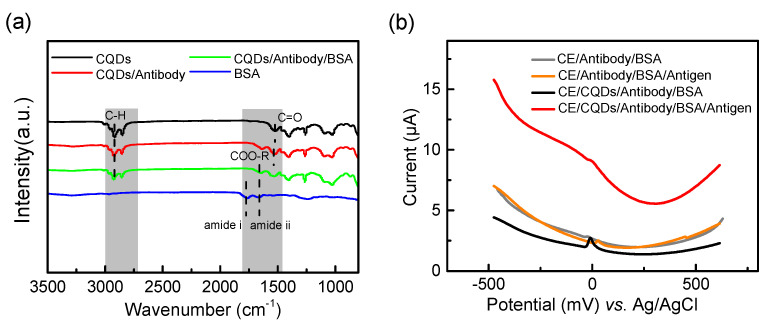
(**a**) FTIR spectra of CQDs, CQDs/Antibody, CQDs/Antibody/BSA and BSA; (**b**) Performance comparison of sensors with CQDs and without CQDs.

Generally speaking, adhesion stability improves with more disease biomarker proteins bound to the sensitive layer of the sensor. Here, FTIR of different samples (Figure 3a) was used to demonstrate that lead sulfide colloidal quantum dots can achieve ligand replacement of MPO antibody proteins by a simple one-step method.

There is an obvious C-H stretching vibration peak in the PbS CQD film at the wave number of 2808~2984 cm^−1^, which originates from the surface oleic acid long-chain ligand carried in the synthesis stage, and the C-H peak of CQD/antibody films at similar positions has been enhanced to a certain extent. However, the carbonyl (C=O) peak of the two samples at the wave number 1526 cm^−1^ position is significantly different, indicating that the surface groups of quantum dots and antibody proteins have changed after cross-linking. Compared with the original PbS CQDs, the carboxylate group (COO-R) replaced the carbonyl group of the OA ligand (C=O). The above results indicated that the target MPO antibody was successfully labeled on CQDs and bound to Pb^2+^ through the O in the carboxylate group of the protein molecule itself. Owing to the large specific surface area and abundant surface suspended bonds, CQDs can stably bind to protein molecules [27]. After the blocking treatment of BSA, the characteristic peak of an amide band can be clearly observed on the curve corresponding to CQD/Antibody/BSA film, indicating that there is stable bovine serum protein on the surface of the electrode. Currently, CQD-based biomolecule detection technology is mainly based on its fluorescence effect [28,29,30]. Yet, the preparation process usually takes hours or even days because of the need for multi-step crosslinking in a liquid-phase environment using the intermediary of a crosslinker [31]. Compared with fluorescent labeling that requires exogenous crosslinking agents, CQDs can achieve one-step replacement of biological protein ligand, resulting in more efficient and cost-effective biochemical sensing.

For immunosensors, efficient signal transduction is the basis for ensuring their sensitivity and specificity. We used differential pulse voltammetry (DPV) in electrochemical detection to compare the MPO antigen detection with and without a CQD-coated electrochemical electrode. The test results are shown in Figure 3b. The results showed that the electrode with a CQD coating could better distinguish the presence of target detection substance MPO. This indicates that PbS CQDs have a prominent signal transduction ability in addition to the function of directly cross-linking proteins. The unique quantum effect of quantum dots could adjust the charge transfer and carrier transport at the interface of CQDs-biomolecules under the effect of an external electric field [32,33], which enables the electrical response to biological immunoreaction.

### 3.2. Electrochemical Characterization of PbS CQD-Modified Electrode

The electrochemical performance of each modified layer was compared. The CQD-modified electrode coated with the bare electrode (CE), PbS CQD-modified electrode (CE/CQDs), and CQD-modified electrode coated with antibody before and after BSA blockade (CE/CQDs/Antibody/BSA) were prepared.

**Figure 4:**
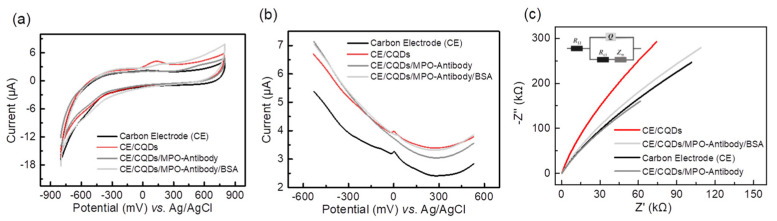
Electrochemical characterizations of the CQD-modified electrode as the MPO sensor. (**a**) CV and (**b**) DPV characterizations of bare carbon electrode (CE), CE/CQDs, CE/CQDs/Antibody, and CE/CQDs/Antibody/BSA in the presence of 1 × PBS. (**c**) The equivalent circuit model of chemically modified electrode and the EIS-fitted curves.

The peak current intensity of the cyclic voltammetry (CV) curve changes with the modification process, indicating the difference in the conductivity of the electrode under the action of different modification layers. The results of CV measurement showed that the above electrodes presented sensitive and reversible REDOX peaks. In addition, the charge transfer ability of different functional modified layers is distinct (Figure 4a). The oxidation peak current of CQD-modified electrode (4.103 μA) was higher than that of the unmodified electrode (2.279 μA). This indicates that the charge transfer ability of the PbS CQD-modified layer was retained, while the change in electrical conduction capacity of the electrode was negligible after protein modification. Differential pulse voltammetry (DPV) can significantly reduce the influence of background current, ensure the high sensitivity and resolution of detection, and is suitable for the detection of low-concentration biochemical molecules. Under the DPV mode test (Figure 4b), the oxidation peak current of the CE/CQDs electrode (3.966 μA) was slightly higher than that of the bare CE electrode (3.261 μA), which was consistent with the CV test results. With the fixation of the protein, the conductivity of the modified electrode decreased slightly. The results above indicate that the CQD-modified layer enhances the interfacial charge transfer from the solution to the solid electrode. EIS can provide quantitative electrical parameters for the equivalent circuit model of the electrochemical system and can be used to analyze the characteristics of the electrode biochemical reaction in the system. Figure 4c illustrates the equivalent circuit model of a quantum dot-modified electrochemical electrode system. It can be seen that although the conductivity of biological protein is poor, the antibody protein BSA and modified electrode are still effective and maintain the ability of charge transduction, and the rule of change is basically consistent with CV and DPV.

### 3.3. Research on the Performance of Immunosensor for Detecting MPO Antigen

**Figure 5:**
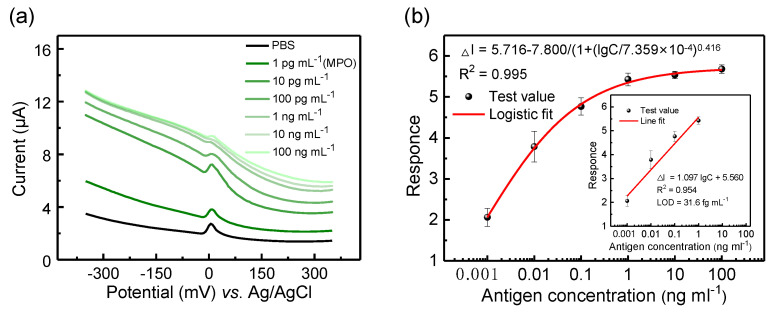
The sensing performance of the PbS CQD-modified electrode for MPO testing. (**a**) DPV curves with the increased antigen concentrations (1 pg mL^−1^ to 100 ng mL^−1^); (**b**) Antigen concentration-dependent response curve using a linear fit.

To investigate the sensitivity of the immunosensor, MPO protein solutions with different concentrations were measured. First, the original sample of MPO antigen was diluted with PBS to obtain different concentrations of MPO protein (1 pg mL^−1^ to 100 ng mL^−1^). In order to study the best test time, the test was carried out every 15 s after dropping the sample, and the test results at different concentrations were consistent. Here, we choose the sample with the concentration of 10 pg mL^−1^ of the target antigen MPO protein as an example to explain the experimental results (Figure S1, Supplementary Material). The test results show that the DPV test current value increases with the passage of test time, and it can be seen that there are two stages in the reaction, in which the sixth test (dripping sample time is 90 s) is the node. Therefore, we choose to read the test results when the antigen–antibody specific binding reaction is 90 s after adding the sample. The measurement range of DPV was 0.8~0.6 V (pulse amplitude = 50 mV; pulse width = 50 ms). As shown in Figure 5a, a peak occurred at around 0 mV~5 mV, and the peak current increased with elevated MPO concentration. In the previous work on ECP detection, the author conducted EIS analysis on the electrochemical detection system and tried to explain the reason for the increase of DPV detection current value with the increase of target protein concentration through the establishment of an equivalent circuit model [26]. We analyze that the working principle of this paper is similar. It may be that with the increase of the concentration of target antigen, the antibody–antigen specific binding reaction at the solid-liquid interface becomes more active, leading to the change of the modified layer on the working electrode, which leads to the decrease of the system film resistance and the enhancement of the charge transduction capacity.

The reference current I_0_ was defined as the peak current of the DPV curve when the MPO antigen concentration was 0, while the sensor response value was defined as the ratio between the test peak current value and I_0_ at different MPO concentrations. Then, the relationship between DPV current response and the logarithm of MPO protein concentration was obtained, and the fitting result was shown in Figure 5b. As shown in Figure 5b, the response signal follows the rule of S-curve response (logarithmic fitting relationship) with the increase of the concentration of target detection protein [34], and the linear fitting is good (R^2^ = 0.995). This result reflects the dynamic process of immune reaction between antigen and antibody; that is, the initial reaction rate is slow. With the increase of antigen concentration, the reaction rate of antigen and antibody binding increases and finally reaches the saturation state. Linear fitting is mainly used to calculate the lower detection limit (LOD). The LOD of the biosensor on MPO antigen was calculated according to the formula (3SD_blank_/slope), which was about 31.6 fg mL^−1^. As shown in Table 1, compared with the reported MPO detection [35,36,37,38,39,40,41], this work demonstrates that the electrochemical sensor has a fast response, low LOD and wide linear dynamic range.

**Figure 6:**
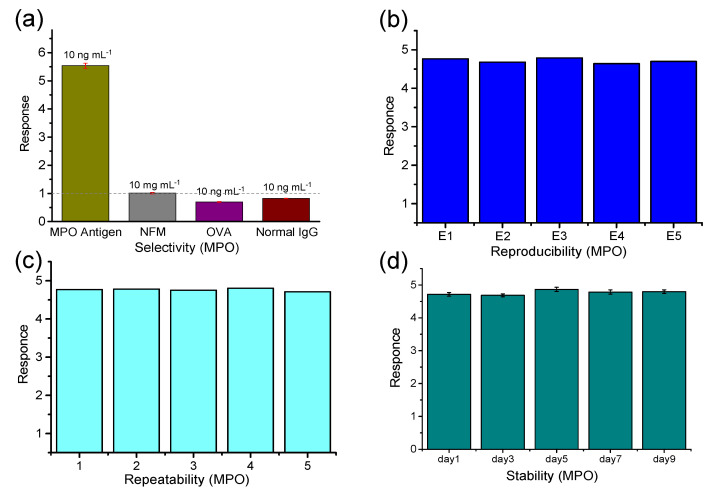
Sensing characteristics: (**a**) Selectivity; (**b**) Reproducibility (single measurement results of five electrodes: E1–E5); (**c**) Repeatability (repeat the measurement five times on one electrode) and (**d**) Stability.

At the same time, the selectivity, repeatability, and stability of the immunosensor for MPO detection were tested, in which the MPO concentration was set as 10 ng mL^−1^. Non-fat dry milk (NFM), ovalbumin (OVA) and human normal IgG were selected to examine the selectivity of the sensor. The detection principle of this work is mainly based on the specific matching reaction between antigen and antibody. With the addition of target antigen, the scanning current of DPV increases, so the response value >1. The addition of other proteins does not bind specifically with the antibody on the surface of the sensor, but causes the resistance of the whole solution system to increase, so the response value <1. As shown in Figure 6a, the responses to NFM, OVA, and IgG with a higher concentration (10 mg mL^−1^) were still much lower than that of MPO (10 ng mL^−1^), which demonstrated good selectivity. To evaluate the reproducibility of the immunosensor, five electrodes were prepared in parallel for comparison (Figure 6b). On the other hand, the test was repeated five times based on one electrode to assess the repeatability (Figure 6c). These results show that intra-sensor and lot-to-batch differences are negligible, demonstrating the potential of the sensors for practical applications. The electrodes were stored in a refrigerator at 4 °C for 9 days and tested with the counter electrode every other day to verify their stability. As shown in Figure 6d, the sensor had good stability under short-term low-temperature storage. Facing the needs of practical application scenarios, it is necessary to optimize the sensor-sealing scheme to meet the long-term storage. The bending experimental results are shown in Figure S2. The experimental results show that bending for about 10 times will hardly affect the detection accuracy of the device.

## 4. Conclusions

In this study, an electrochemical immunosensor based on PbS CQDs was developed to detect MPO with high sensitivity. The colloidal quantum dot semiconductor nanocrystals were modified on the surface of the planar three-electrode to form a flexible amperometric immunosensor, which enables the electrical response to biological immunoreaction. The flexible and portable biosensor has a wide detection range from 1 pg mL^−1^ to 1 ng mL^−1^, as well as high sensitivity (LOD = 31.6 fg mL^−1^). Nice selectivity, reproducibility, reliable repeatability, and short-term storage stability in low temperature were demonstrated in this work. The accurate MPO detection enabled by this biosensor can be combined with early ECP detection, which is significant for the precise diagnosis of the nature and degree of the local inflammation in rhinitis treatment. The colloidal quantum dot immunoassay technology developed in this work has broad application prospects in the development of low-cost, high-throughput clinical diagnostic instruments, POCT, home detection and other aspects.

## Figures and Tables

**Table 1:** Parameters comparison for the detection of MPO biomarkers.

Modified Electrode	Measurement Mode	LOD (pg mL^−1^)	Linear Range (ng mL^−1^)	Reference
ITO-nano-Au/PoPD-MWCNTs-IL	CV	50	23.4–300	[35]
IrOx-TiN	EIS	500	1–1000	[36]
Au-PoPD-MWCNTs/Au	CV	70	0.25–350	[37]
CE-MPs/CNT	CV	543	0–120	[38]
Au-L-Cysteine/BMIMPF6/CeO2	CV	60	10–400	[39]
N-CNT	-	700	100–210	[40]
Graphene-coated IDE-arrays	-	350	0.112–3000	[41]
CE-PbS CQDs	DPV	0.0316	0.001–1	This Work

## Data Availability

Not applicable.

## Supplementary Materials

The following supporting information can be downloaded at: https://www.mdpi.com/article/10.3390/bios13020255/s1, Figure S1: DPV scanning curve versus time at 10 pg ml^−1^ MPO detection concentration (test every 5 s); Figure S2: Experimental results of device bending test.
