# Supplementary material for: Flexible Amperometric Immunosensor Based on Colloidal Quantum Dots for Detecting the Myeloperoxidase (MPO) Systemic Inflammation Biomarker
Source: Biosensors (Basel). 2023 Feb 10;13(2):255. doi: 10.3390/bios13020255 (PMC9954662; doi:10.3390/bios13020255)
Supplement: Supplementary file 1 [file biosensors-13-00255-s001.zip › biosensors-2067073-supplementary.pdf]

Supplementary File(s)

The antigen-antibody binding reaction is phased and can be divided into two stages. The first stage is the specific binding stage of antigen and antibody. This stage is the complementary non-covalent binding between antigen and antibody. The reaction is rapid and can be completed in seconds to minutes. The second stage is the visible reaction stage, which is the process that small antigen-antibody complexes attract to form larger complexes by positive and negative charges. The reaction is slow at this stage, and the time required varies from minutes, hours to days.

In order to study the best test time, the test was carried out every 15 seconds after dropping the sample, and the test results at different concentrations were consistent. Here, we choose the sample with the concentration of 10 pg ml^{-1} of the target antigen MPO protein as an example to explain the experimental results (Fig. S1). The test results show that the DPV test current value increases with the passage of test time, and it can be seen that there are two stages in the reaction, in which the sixth test (dripping sample time is 90s) is the node. Therefore, we choose to read the test results when the antigen-antibody specific binding reaction is 90s after adding the sample.

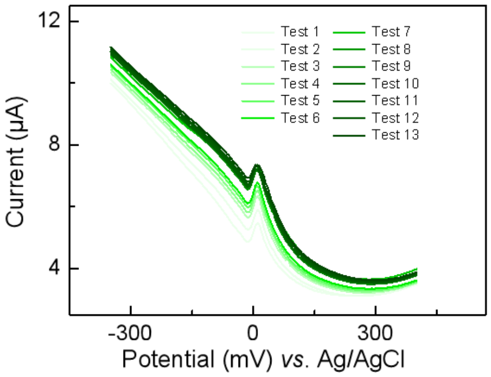

Figure S1. DPV scanning curve versus time at 10 pg ml^{-1} MPO detection concentration (test every 5s).

We tested and compared the devices after bending, and the experimental results are shown in Figure S2. The experimental results show that bending for about 10 times will hardly affect the detection accuracy of the device.

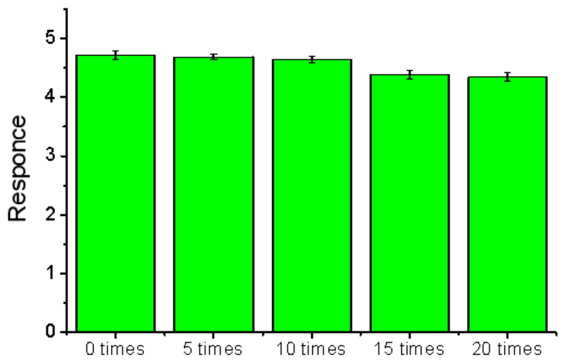

Figure S2. Experimental results of device bending test.
